# Changes in macrophage-like cells characterized by en face optical coherence tomography after retinal stroke

**DOI:** 10.3389/fimmu.2022.987836

**Published:** 2022-09-13

**Authors:** Yunkao Zeng, Feng Wen, Lan Mi, Yuying Ji, Xiongze Zhang

**Affiliations:** State Key Laboratory of Ophthalmology, Zhongshan Ophthalmic Center, Sun Yat-sen University, Guangdong Provincial Key Laboratory of Ophthalmology and Visual Science, Guangzhou, China

**Keywords:** macrophage-like cell, stroke, central retinal artery occlusion (CRAO), optical coherence tomography, neuroinflammation, microglia

## Abstract

**Purpose:**

The retina could serve as a window of neuroinflammation, but the *in vivo* changes in macrophage-like cell (MLC), such as microglia, in acute ischemic retinal stroke remain unclear. Thus, the current study aimed to investigate the *in vivo* changes in MLC characterized by en face optical coherence tomography (OCT) after acute ischemic retinal stroke.

**Methods:**

Twenty patients with unilateral acute nonarteritic reperfused central retinal artery occlusion (CRAO) were participated in this study, and their contralateral eyes served as control group. A 3 μm en face OCT slab on the inner limiting membrane of the optic nerve head (ONH) region or macular region was used to visualize and binarize the MLCs. The MLCs were binarized and quantified using a semiautomated method. OCT angiography was used to evaluate the reperfusion status and obtain the structural data of the inner retina in the ONH and macula. The thickness of the ganglion cell complex in the macular region was measured. The optical intensity and optical intensity ratio of the inner retina were calculated to evaluate the ischemia severity.

**Results:**

In the ONH region, decreased vessel densities of radial peripapillary capillaries accompanied by increased thickness of the retinal nerve fiber layer were found in the CRAO eyes in comparison to the unaffected eyes (p=0.001, p=0.009, respectively). In the macular region, significantly lower vessel densities in both the superficial and deep capillary plexus and increased thickness of the ganglion cell complex were also found in the CRAO eyes (all p ≤ 0.001). The ONH and macular MLC quantities and densities in CRAO eyes were significantly higher than those in the unaffected eyes (both p<0.001). Larger and plumper MLCs were observed in the CRAO eyes compared with their unaffected eyes. ONH and macular MLC densities were positively associated with the disease duration in the acute phase and the optical intensity ratio of inner retina.

**Conclusions:**

The increased density and morphological changes of MLCs may indicate the aggregation and activation of MLCs following acute reperfused CRAO. The aggregation of MLCs may be more pronounced in CRAO eyes with longer disease duration and more severe ischemia. MLCs characterized by en face OCT may serve as an *in vivo* visual tool to investigate neuroinflammation in the ischemic-reperfusion process of stroke.

## Introduction

Retinal stroke refers to central retinal artery occlusion (CRAO) caused by compromised blood flow *via* the central retinal artery to the inner retina (1). The estimated age- and sex- adjusted incidence of acute CRAO is 1-2.5 per 100,000 persons per year (2–5). Timely recanalization of the occluded artery to achieve reperfusion and salvage damaged neurons is the main goal of most interventions. Infarction of the inner retina, although mostly reperfused, may still lead to permanent visual loss mainly caused by secondary reperfusion injury following blood supply reconstruction (6). Only 17% of patients with CRAO achieve functional visual acuity in the affected eye (1). Ischemia-reperfusion injury is a common feature and a therapeutic challenge of ischemic stroke. To date, the lack of an effective neuroprotective strategy in ischemic stroke may partially be due to gaps between experimental and clinical studies (7). As part of the central nervous system, the retina shares a similar pathologic process with the brain in acute ischemic stroke (1, 8–10). Therefore, as a visible neural tissue, the retina could be a perfect window to evaluate the *in vivo* cellular response of neural tissue in the ischemia-reperfusion injury of stroke.

Increasing amounts of research data from clinical and experimental stroke studies have revealed that neuroinflammation following ischemia-reperfusion injury is an essential component for the subsequent pathophysiological process. Specifically, previous studies found that resident immune cells such as microglia and peripheral monocyte-derived macrophages play essential roles in modulating the progression of neuroinflammation in stroke (11, 12). Microglia are regarded as prototypic tissue-resident macrophage-like innate immune cells in the central nervous system (13). Macrophage-like cells (MLC) candidates include microglia, perivascular macrophages, monocyte-derived macrophages, and hyalocytes from vitreous (14). Despite the key roles they play in neuroinflammation, MLCs have been studied predominantly *ex vivo* or in animal models (13).

Currently, the advance of label-free adaptive optics optical coherence tomography (OCT) and en face OCT allows us to visualize human retinal MLCs near the inner limiting membrane (15, 16). *In vivo* research on MLCs in living nervous tissue is limited in patients with stroke. Investigating the impact of acute retinal ischemia and reperfusion on MLCs may provide further insight into the pathophysiological process of neuroinflammation following artery occlusion. The inner limiting membrane is an extremely thin layer of the inner retina, which covers the ganglion cell complex or retinal nerve fiber layer. Previous studies revealed that MLCs on the inner limiting membrane played important roles in parainflammation or neuroinflammation in chronic neurodegenerative or chronic neurovascular disease (17, 18). We propose that MLCs may undergo quantitative and morphological changes in the ischemia-reperfusion process caused by acute arterial occlusion. The current study aimed to investigate the impact of acute ischemia and reperfusion on MLCs *in vivo* using en face OCT and identify the potential clinical significance of MLCs in retinal stroke.

## Methods

### Subjects

This was a cross-sectional study involving patients with nonarteritic unilateral reperfused CRAO at their first visit between August 2021 and March 2022 to Zhongshan Ophthalmic Center, China. Patients in the acute phase with a disease duration ≤15 days were included in the study. The unaffected eyes served as the control group. The current study adhered to the tenets of the Declaration of Helsinki and was approved by the Institutional Review Board of the Zhongshan Ophthalmic Center. Written informed consent was obtained from all included subjects. All patients underwent a complete ocular examination, including intraocular pressure, refractive error (autorefractometry), best-corrected visual acuity (LogMAR), slit-lamp fundus examination, OCT angiography and en face OCT examination. The exclusion criteria were as follows (1): patients with refractive error > 3 diopters[D] (2);. patients with any other ocular inflammatory retinal and choroidal disease (uveitis, white dot syndrome, etc.) (3); history of other ocular vascular diseases (4); history of ocular trauma, ocular surgery, or steroid treatment, and (5) OCT angiography and en face OCT images with poor quality (scan quality <6 or obvious artefacts).

### Measurement of neurovascular parameters and status of reperfusion

OCT angiography (RTVue Avanti; Optovue, Fremont, CA, USA) was used to evaluate the reperfusion status and structural data of the retina in the optic nerve head (ONH) region and macular region. A high density (HD) disc 4.5×4.5 mm program was used to measure the vessel density of radial peripapillaries capillary and the thickness of the retinal nerve fiber layer in the ONH region. The HD macular 6×6 mm program was used to measure the vessel density of the superficial capillary plexus, and deep capillary plexus in the macular region and obtained the structural data of macular region.

All OCT/OCTA examinations were completed by the same experienced examiner (Y.Z.). Patients with poor vision and fixation were asked to stare at an external fixation light with the unaffected eye. We also turned on the eye tracking function of the device to reduce motion artefacts. The examinations were conducted under standardized procedures provided by the manufacturer. To achieve better imaging quality, two orthogonal OCT angiography volumes were obtained at the same location and then registered using a motion correction algorithm (19). In addition, the HD mode also ensured high quality and high definition of images in our study. The HD mode is capable of enhancing the signal to noise ratio and reducing image artefacts, since it gathers more data points from the retina and leads to higher vertical, horizontal, and axial resolution (20). The OCT angiography images, en face OCT images and B scan OCT images were automatically generated after examination. All the images were checked for scan quality and artefacts after obtaining the data. If the scan quality was lower than 6 or obvious artefacts were found, the examiner reexamined the patient. If the scan quality was still lower than 6 or obvious artefacts were still present after repeating the exam twice, patients were excluded from the study. The vessel densities, average thickness of the macular ganglion cell complex layer, and average retinal nerve fiber layer were automatically calculated by the inbuilt software (software version 2017.1.0.155). The ganglion cell complex in the macular region was defined as the innermost three layers in the retina, including the nerve fiber layer, ganglion cell layer and inner plexiform layer (21).

### Measurement of optical intensity

Optical intensities and optical intensity ratios of the inner retina on OCT in the ONH and macular region were calculated to evaluate the ischemia severity (22), since the ischemic oedema of the inner retina increased its optical density correspondingly during CRAO. Optical density was defined as the mean reflectivity of the retinal layers on OCT images. Optical intensity could be affected not only by the reflectivity of the tissue, but also by other confounding factors, such as signal strength, and the power of the OCT laser light (23). Thus, the optical intensity ratio was used to normalize the effect of confounding factors (22). The horizontal OCT B scan across the center of the ONH or fovea centralis was exported as greyscale JPEG images from the abovementioned system. The optical intensity is the average grey level of selected regions on a scale of 0 (pure black) to 255 (pure white) and was automatically calculated by ImageJ software (22–24). The optical density of the inner retina (including the retinal nerve fiber layer, ganglion cell layer, inner plexiform layer, inner nuclear layer, and outer plexiform layer) and the vitreous cavity was calculated with a modified method as in previous studies (22, 25). The optical intensity ratio of the inner retina was defined as the optical intensity of the inner retina over the optical intensity of the vitreous cavity.

### Measurement of MLC parameters

To visualize the MLCs on the inner limiting membrane, a 3 μm slab in the ONH and macular region was manually adjusted as previously described (16). The retinal nerve fiber layer located from 0 μm to 28 μm below the inner limiting membrane was also segmented. Using the 3 μm slab and 28 μm slab, the MLCs were isolated and quantified using a semiautomated binarization process with ImageJ (24) as previously described in a masked fashion (18). The main processes include (1): noise reduction to remove background irregularities and vessel artifacts (2); signal enhancement to improve MLC identification; and (3) binarization to extract cell shapes. Finally, the analyzed particles function of ImageJ was used to calculate the number of MLCs from binarized images. The density of MLCs was also calculated.

### Statistical analysis

SPSS software version 19.0 (SPSS Inc, Chicago, IL) was used to analyze the data. The Shapiro–Wilk test was performed to test the normality of the data. Continuous variables with a normal distribution are presented as the mean ± standard deviation. Nonnormal variables are reported as the median (interquartile range). Categorical variables are reported as frequencies. The differences in vessel density, ganglion cell layer thickness, retinal nerve fiber thickness, optical intensity, and optical intensity ratio between CRAO eyes and fellow eyes were compared using a paired t test. The differences in the number and density of MLCs between CRAO eyes and fellow eyes were compared using the Wilcoxon signed rank test. Stepwise multiple linear regression analysis was used to test the independent association of each variable with the MLC density. A two-sided p < 0.05 was considered statistically significant.

## Results

After excluding two patients with poor image quality, 40 eyes of 20 unilateral nonarteritic CRAO patients were included in the study. The unaffected 20 fellow eyes served as the control group. The mean age of the included patients was 49.60 ± 13.37 years, and half of them were female (10/20). The duration of disease at the time of examination was 5.95 ± 4.31 days, ranging from 1 to 15 days. The best corrected visual acuity (LogMAR) of the CRAO eyes was far worse than that of their unaffected fellow eyes (2.030 ± 0.74 vs. -0.005 ± 0.06, p<0.001).

In terms of the optical intensity ratios of the inner retina, results are shown in **Table 1**. Specifically, the ONH optical intensities of the inner retina in CRAO eyes were higher than those in the unaffected fellow eyes (143.46 ± 7.81 vs. 132.06 ± 8.73, p<0.001). The vitreous optical intensity above the ONH region was comparable between the CRAO eyes and their fellow eyes (15.21 ± 0.81 vs. 15.11 ± 1.27, p=0.775). The ONH optical intensity ratio of the inner retina was significantly elevated in eyes with CRAO (9.45 ± 0.63 vs. 8.79 ± 0.86, p=0.001). In the macular region, the optical intensities of the inner retina and vitreous in CRAO eyes were higher than those in the unaffected fellow eyes (138.26 ± 16.89 vs. 103.58 ± 9.94, p<0.001 and 15.30 ± 2.20 vs. 13.61 ± 1.81, p<0.001 respectively). The macular optical intensity ratio of the inner retina was significantly elevated in eyes with CRAO (9.27 ± 1.22 vs. 7.72 ± 1.08, p<0.001).

**Table 1:** The optical intensity on optical coherence tomography image of the CRAO eyes and fellow eyes.

	CRAO eyes (n = 20)	Fellow eyes (n = 20)	p value
**ONH region**			
Inner retina	143.46 ± 7.81	132.06 ± 8.73	<0.001^†^
Vitreous	15.21 ± 0.81	15.11 ± 1.27	0.775^†^
Optical intensity ratio	9.45 ± 0.63	8.79 ± 0.86	0.001^†^
**Macular region**			
Inner retina	138.26 ± 16.89	103.58 ± 9.94	<0.001^†^
Vitreous	15.30 ± 2.20	13.61 ± 1.81	<0.001^†^
Optical intensity ratio	9.14 ± 1.22	7.72 ± 1.08	<0.001^†^

CRAO, central retinal artery occlusion; ONH, optic nerve head; †data was described as the mean ± standard deviation and compared using paired T test.

In terms of other neurovascular parameters and MLC parameters, the detailed data and comparisons are shown in **Table 2**. In the ONH region, the radial peripapillary capillary vessel density was significantly decreased in CRAO eyes compared to fellow eyes (43.27 ± 5.05 vs. 48.94 ± 4.82, p=0.001). In contrast, the thickness of the retinal nerve fiber layer was significantly increased in the CRAO eyes in comparison to the fellow eyes (139.4 ± 36.40 μm vs. 119.10 ± 18.97 μm, p=0.009). In the macular region, both vessel densities of the superficial capillary plexus and deep capillary plexus were significantly lower in the CRAO eyes than in the fellow eyes (42.69 ± 6.54 vs. 48.24 ± 3.84, p<0.001; and 41.45 ± 6.21 vs. 47.27 ± 4.79, p<0.001, respectively). Conversely, the ganglion cell complex thickness of the CRAO eyes was significantly higher than that of their fellow eyes (126.30 ± 37.39 μm vs. 96.75 ± 11.27 μm, p=0.001).

**Table 2:** MLCs parameters and retinal neurovascular parameters in the CRAO eyes and fellow eyes.

	CRAO eyes (n = 20)	Fellow eyes (n = 20)	p value
**ONH region**			
RPC VD	43.27 ± 5.05	48.94 ± 4.82	0.001^†^
RNFL thickness (μm)	139.4 ± 36.40	119.10 ± 18.97	0.009^†^
ONH MLC count	493.50 (418.00, 652.75)	330.00 (246.50, 423.75)	<0.001^‡^
ONH MLC density (cells/mm^2^)	24.37 (20.64, 32.23)	16.27 (12.17, 20.93)	<0.001^‡^
**Macular region**			
Macular VD in SCP	42.69 ± 6.54	48.24 ± 3.84	<0.001^†^
Macular VD in DCP	41.45 ± 6.21	47.27 ± 4.79	<0.001^†^
GCC thickness (μm)	126.30 ± 37.39	96.75 ± 11.27	0.001^†^
Macular MLC count	553.50 (471.00, 752.50)	274.00 (176.25, 339.75)	<0.001^‡^
Macular MLC density (cells/mm^2^)	15.38 (13.08, 20.90)	7.61 (4.90, 9.44)	<0.001^‡^

MLC, macrophage-like cell; CRAO, central retinal artery occlusion; ONH, optic nerve head; RPC, radial peripapillary capillary; VD, vessel density; RNFL, retinal nerve fiber layer; SCP, superficial capillary plexus; DCP, deep capillary plexus; GCC, ganglion cell complex; †data was described as the mean ± standard deviation and compared using paired T test; ‡data was described as median (interquartile range) and compared using the Wilcoxon signed rank test.

**Figure 1:**
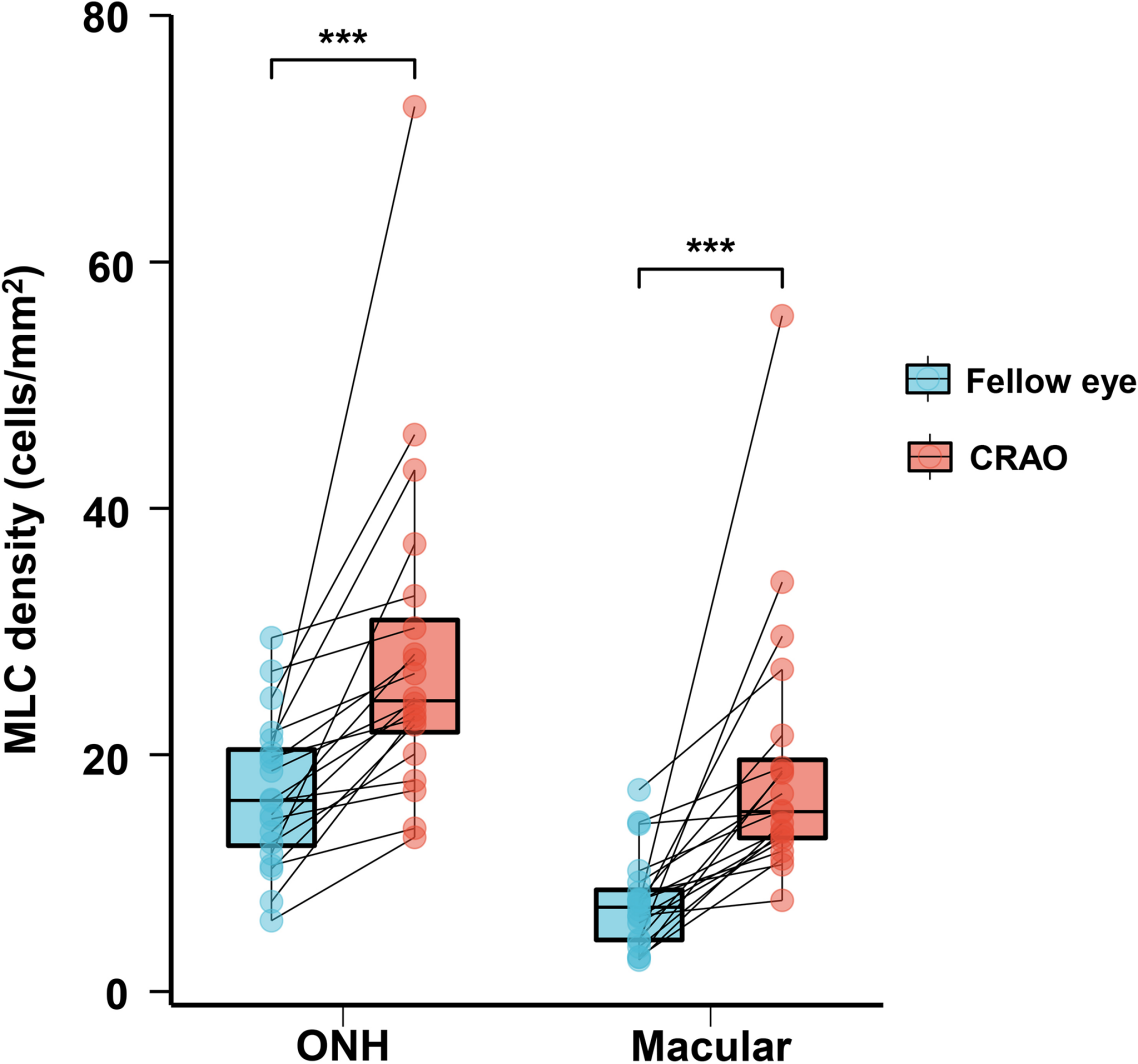
The data distribution and comparison of ONH and macular MLC densities between unaffected fellow eyes and CRAO eyes. The ONH and macular MLC densities were significantly higher in CRAO eyes. MLC, macrophage-like cell; CRAO, central retinal artery occlusion; ONH, optic nerve head; ***p<0.001.

**Figure 2:**
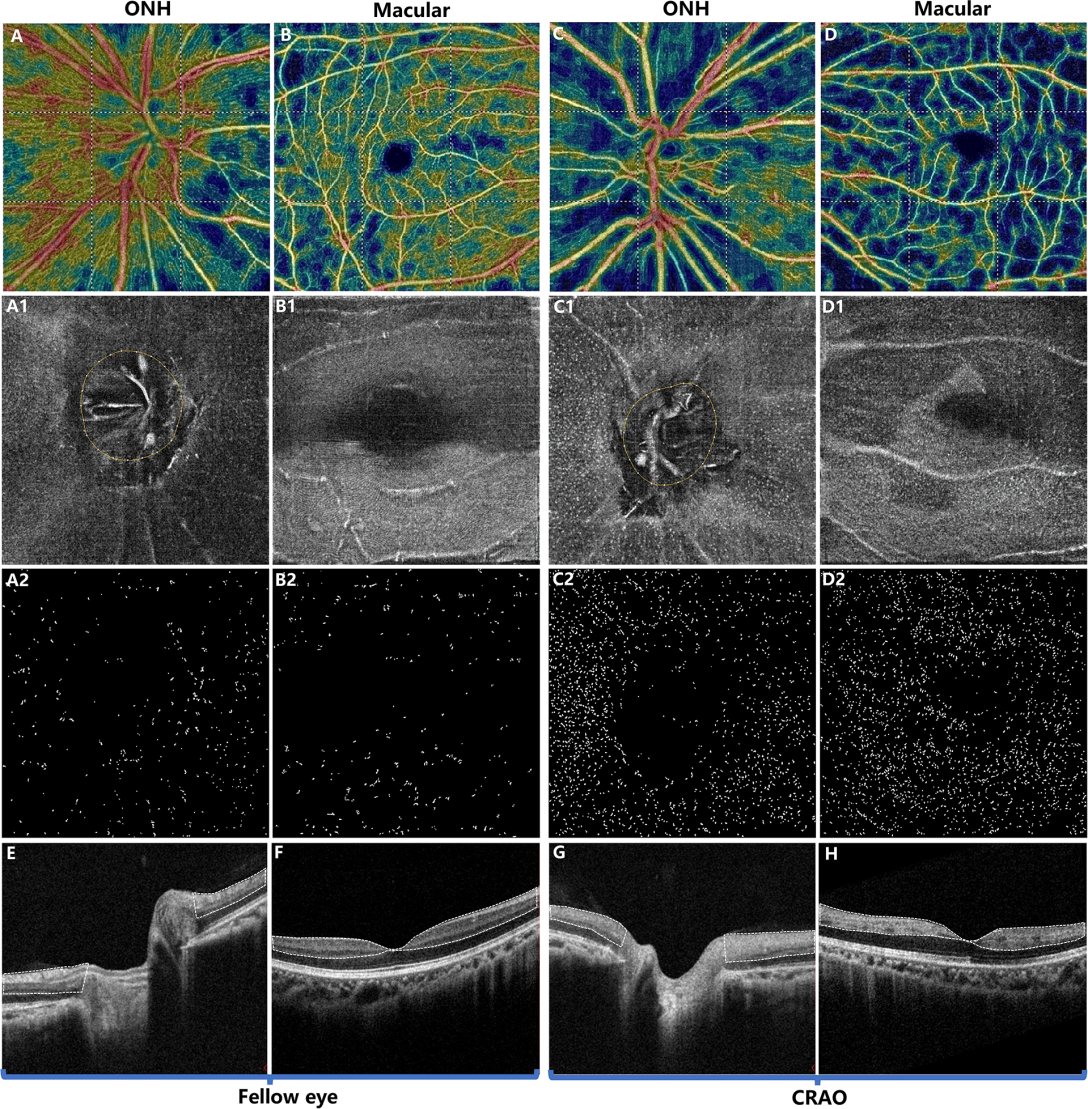
Typical example of a patient with acute nonarteritic CRAO for 15 days. The first and third columns show the images of the ONH region while the second and fourth columns show the images of the macular region. The first row **(A–D)** shows the OCT angiography images. The second row **(A1–D1)** shows the 3 μm en face OCT slabs, and the third row **(A2–D2)** shows the extracted MLCs from **(A1–D1)**. Compared to the unaffected fellow eye **(A, B)**, the radial peripapillary vessel density **(A, C)** and macular vessel density **(B, D)** were decreased in CRAO eyes **(C, D)**. In contrast, the ONH and macular MLCs were more abundant in CRAO eyes **(C1, C2, D1, D2)** than in the fellow eyes **(A1, A2, B1, B2)**. **(E, F)** OCT showing normal retina structures and normal optical intensity of the inner retina (the region circled by the dashed line). **(G, H)** OCT of CRAO eyes showing increased inner retinal optical intensity (the region circled by dash line), indicating inner retinal ischemia and edema. MLC, macrophage-like cell; CRAO, central retinal artery occlusion; ONH, optic nerve head; OCT, optical coherence tomography.

**Figure 3:**
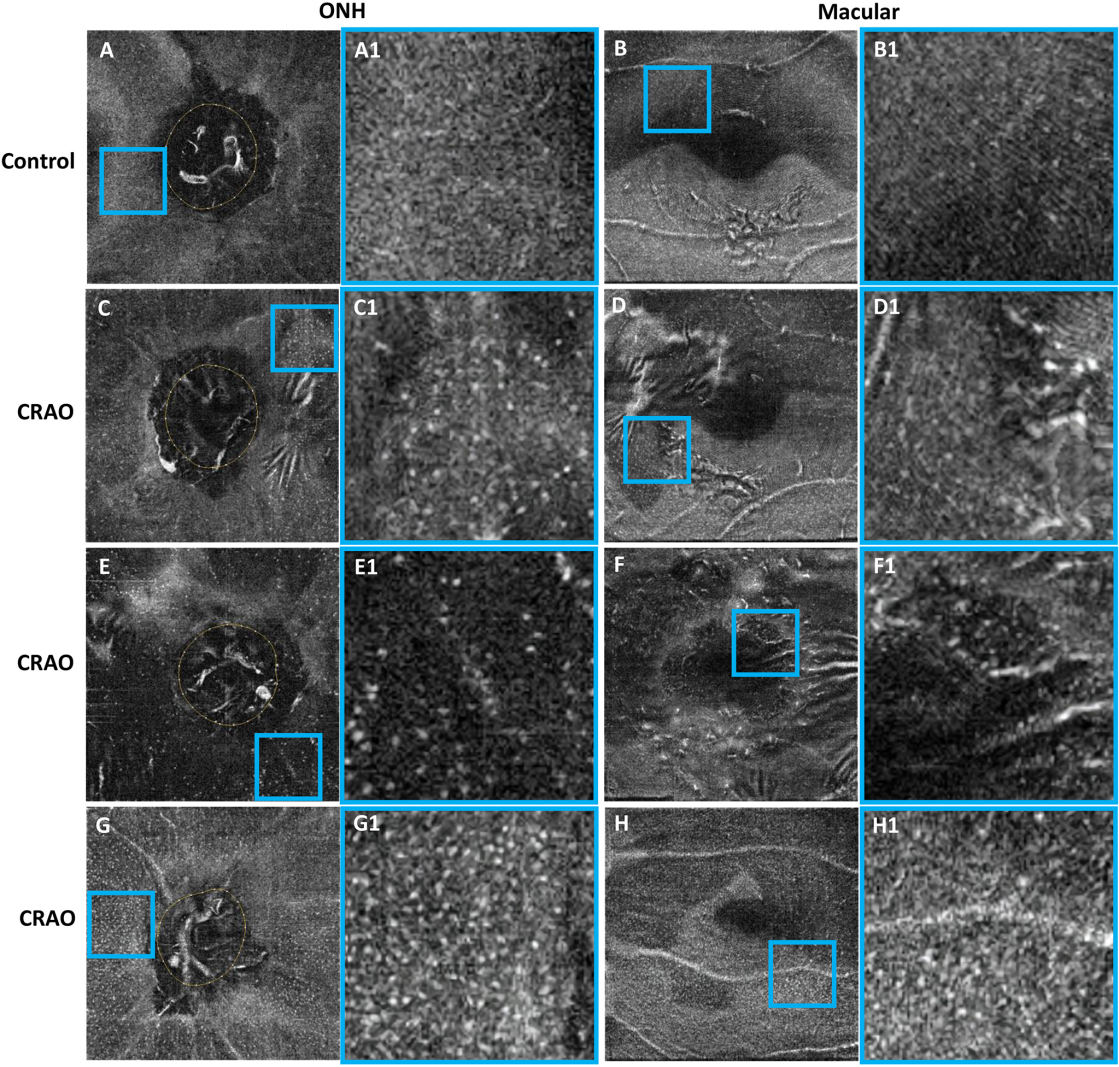
Typical examples showing the cellular morphology in a normal eye (first row), its contralateral eye with CRAO (second row) and two CRAO eyes from another two patients (third row and fourth row). The first and third columns show the 3 μm en face OCT of the ONH and macular region **(A–H)**, respectively. The regions of interest (ROIs) in the ONH region and macular region are indicated by blue boxes. The ROI is enlarged by 4 times and shown on the right side of the original en face OCT **(A1–H1)**. The MLCs appear larger and rounder with fewer protrusions in eyes with CRAO. MLC, macrophage-like cell; CRAO, central retinal artery occlusion; ONH, optic nerve head; OCT, optical coherence tomography.

With regard to MLC parameters, the numbers of ONH and macular MLCs in CRAO eyes were significantly increased in comparison to fellow eyes (median (interquartile range): 493.50 (418.00, 652.75) vs. 330.00 (246.50, 423.75), p<0.001 and 553.50 (471.00, 752.50) vs. 274.00 (176.25, 339.75), p<0.001, respectively). Similarly, the ONH and macular MLC densities in CRAO eyes were also significantly increased in comparison to fellow eyes (median (interquartile range): 24.37 (20.64, 32.23) cells/mm^2^ vs. 16.27 (12.17, 20.93) cells/mm^2^, p<0.001 and 15.38 (13.08, 20.90) cells/mm^2^ vs. 7.61 (4.90, 9.44) cells/mm^2^, p<0.001, respectively). **Figure 1** shows the data distribution and comparison of MLC density between CRAO eyes and fellow eyes. We found that the distribution of MLCs was scattered without obvious perivascular clustering or gathering. A typical example is shown in **Figure 2**. Along with the increased quantity and density of MLCs, changes in MLC morphology were also observed on the inner limiting membrane of CRAO eyes. In the unaffected fellow eyes, the MLCs appeared slender with spindle- or star-like configuration. However, their morphology transformed into a larger and rounder appearance with fewer protrusions in eyes with CRAO (**Figure 3**).

**Figure 4:**
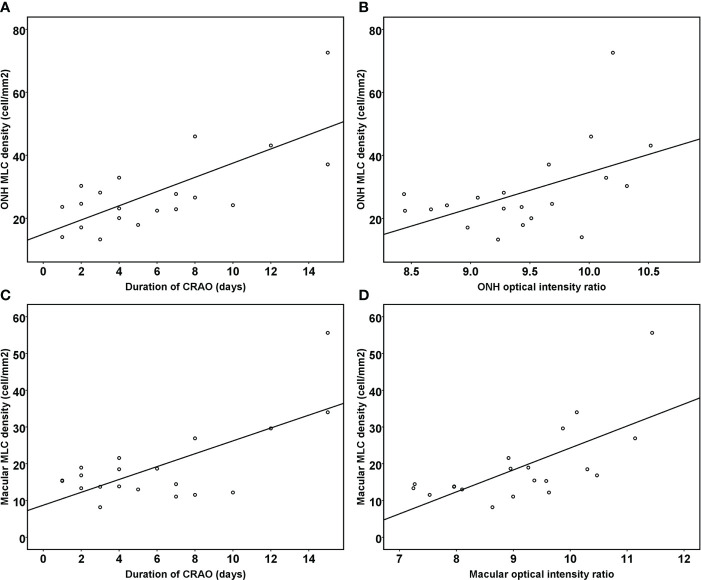
Scatter plots showing the correlation between the duration of reperfused CRAO, the optical intensity ratio of the inner retina and MLC density. The ONH MLC density was positively associated with the duration of disease course **(A)** and the ONH optical intensity ratio of the inner retina **(B)** (R^2^ **^=^** 0.702, p<0.001). The macular MLC density was also positively associated with the duration of disease course **(C)** and the macular optical intensity ratio of the inner retina **(D)** (R^2^ **^=^** 0.642, p<0.001).

The results of stepwise multiple linear regression analysis are shown in **Table 3**. The age, disease duration, radial peripapillary vessel density, retinal nerve fiber thickness and ONH optical density ratio of the inner retina were entered into a regression model to test the independent factors associated with ONH MLC density. Similarly, age, disease duration, macular vessel density in the superficial capillary plexus, macular vessel density in the deep capillary plexus, GCC thickness and macular optical density ratio of inner retina were entered into a regression model to test the independent factors associated with macular MLC density. The results revealed that both the disease duration and optical intensity ratio of the inner retina were positively correlated with MLC density (**Figure 4**).

**Table 3:** Stepwise multivariate linear regression analyses of factors associated with MLC density in CRAO eyes.

	β	95% CI	standardized β	p value
**ONH region**				
Optical intensity ratio of inner retina	2.089	(1.206, 2.971)	0.666	<0.001
Disease duration	9.564	(3.366, 15.753)	0.434	0.005
R^2 =^ 0.702				
**Macular region**				
Optical intensity ratio of inner retina	4.185	(1.403,6.966)	0.474	0.006
Disease duration	1.281	(0.493,2.069)	0.512	0.003
R^2 =^ 0.642				

MLC, macrophage-like cell; CRAO, central retinal artery occlusion; ONH, optic nerve head.

## Discussion

In the current study, we investigated the *in vivo* changes in MLCs in the ischemia-reperfusion retina caused by acute artery occlusion. To the best of our knowledge, this is the first study to visualize the MLCs responding to ischemia-reperfusion injury in living human retina. In eyes with acute reperfused CRAO, increased density and changes in the morphology of MLCs were found, which may indicate the activation and aggregation of MLCs. Moreover, MLCs were more abundant in eyes with longer disease durations and more severe ischemia.

The inner retinal optical density and optical intensity ratio are reliable indicators of ischemia in patients with CRAO (23, 25). Moreover, the vessel density detected by OCT angiography may partially reflect the reperfusional status of the retina. Retinal stroke is a process of acute ischemia and reperfusion. Following acute occlusion of the central retinal artery, blood flow slows down dramatically and the capillary supplying the inner retina starts to collapse. Reduced blood supply to the cells resulted in intracellular edema, leading to increased thickness of the ganglion cell complex, retinal nerve fiber layer, and optical intensity ratio of the inner retina. After recanalization of the occluded artery, the blood flow was gradually restored. Such changes could be detected by OCT angiography as decreased vessel density in the ONH and macular region in CRAO eyes. However, reperfusion of the inner retina could not reverse the existing ischemic changes and may cause further secondary damage probably by the MLC alterations we observed in this study.

There are two kinds of specific resident macrophages on the normal inner limiting membrane: hyalocyte and microglia (15). A previous study revealed distinct differences between hyalocytes and monocytes, but a high degree of similarity was observed between these two cells (26). Although we could not differentiate microglia and hyalocytes based on label-free en face OCT, cumulative evidence has shown that both cells undergo proliferation and activation in neuroinflammation (12, 27, 28). In fact, retinal microglia are located primarily in the inner plexiform layer and outer plexiform layer under normal conditions, and their quantity is relatively lower on the inner limiting membrane (28). Similarly, the number of hyalocytes in the vitreous is low under physiological conditions (29). In addition, they are located at an average distance of 50 mm from the inner limiting membrane and are primarily located anteriorly at the vitreous base and posteriorly in the vicinity of the optic nerve head (27). Therefore, the quantity of MLCs, such as hyalocytes and microglia in the 3 μm en face OCT slab on the inner limiting membrane is quite low in normal eyes. This OCT slab provides a low signal background for visualizing MLCs, through which to observe the total changing profile of MLCs in retina.

Recently, MLCs on the inner limiting membrane in mice were confirmed to be self-renewing cells and predominantly microglia with minor populations of perivascular macrophages and vitreal hyalocytes at steady state, but predominantly monocytes and monocyte-derived macrophages under neuroinflammation (30). We and others have demonstrated signs of aggregation and activation of MLCs following retinal stroke, retinal vein occlusion, diabetic retinopathy and Behçet’s uveitis (17, 18, 31). The density of MLC well indicates the extent of fluorescein leakage and the status of inflammation in Behçet’s uveitis (31). These findings suggest that MLCs on the inner limiting membrane characterized by en face OCT are a potential biomarker for retinal neuroinflammation in clinical practice, which might indicate the required anti-inflammatory treatment and facilitate personalized medicine. However, further longitudinal observational and interventional studies should be conducted to investigate the roles and clinical significance of MLC. Given that the eye has great advantages on observing both the neurovascular and immune cell changes in ischemic stroke, the retina could serve as a great tool in translational stroke study.

In response to neural damage, local microglia/macrophages become activated with changes in morphology, proliferation, and migration to the damaged areas (32–35). Following activation, microglia retract their ramified protrusions and enhance their phagocytic capacity to clear debris and cellular corpses, which may further change their size and roundness (36, 37). Activated microglia can also alter the blood-brain barrier to recruit circulating immune cells, such as macrophages, to exacerbate brain injury after cerebral stroke (38, 39). In a rodent retinal ischemia-reperfusion model induced by elevated intraocular pressure, microglia/macrophages were activated in an increased number not only by the migration and proliferation of resident microglia, but also by the recruitment of circulating monocytes (35). We observed that the MLCs in human retinal stroke after reperfusion increased in number and density and became more amoeboid in morphology. This is in accordance with findings from the above studies in cerebral stroke or ischemic retinal animal experiments. It is difficult to distinguish activated microglia from activated macrophages morphologically following stroke (11, 40). Thus, the MLCs characterized by en face OCT could also comprise other immune cells recruited from blood circulation. During neuroinflammation in mouse model, Ly6C+ monocytes and monocyte-derived macrophages are significant additional components of the heterogenous vitreoretinal interface macrophage population (30). Recruited MLCs might migrate to ischemic retina, observed in a scattered distribution without obvious perivascular clustering or gathering. In this study, the increased MLC density in reperfused retinal stroke was associated with disease duration and ischemia severity, but not with reperfusional status of the retina. In the nonperfused area of the retina in diabetic retinopathy and retinal vein occlusion, although suffering more severe ischemia compared to the perfused area, the MLC density is relatively low (17, 18). Reperfusion seems to be a prerequisite for MLC increase by providing the channel for MLC recruitment from circulation and the support for MLC survival. After reperfusion, ischemia severity and disease duration might determine the extent of MLC aggregation and activation. In an animal model of cerebral stroke, the shorter occlusion time and less tissue damage also result in a lower macrophage cell density, while the quantity of macrophages/microglia continued to increase until Day 4 to Day 7 after reperfusion and then reached a plateau for a longer period (41).

However, the release of a wide range of toxic and proinflammatory mediators by activated microglia/macrophages can be harmful and worsen retinal disease (32, 33, 37). In a rodent retinal ischemia-reperfusion model, microglia/macrophage activation was associated with secondary apoptotic retinal ganglion cells after reperfusion and started days before the later onset of structural damage (42). Therefore, MLCs might be a potential target for neuroprotective treatment in ischemia-reperfusion injury.

There are several limitations in this study. First, this is a cross-sectional study involving patients at their first visit to avoid the interference of subsequent treatment. Further prospective studies investing the consecutive changes of MLC changes is needed. Second, we did not register repeated en face OCT images to enhance the image quality according to previous studies (16, 18). For patients with low vision and poor fixation, such as CRAO patients, redundant examination is time-consuming and prone to introduce novel artifacts and buildup of noise signals, which affects the accuracy of detecting MLCs. We isolated and quantified MLC using single en face OCT imaging with HD mode in this study and our previous study (17, 31). We were trying to provide a timesaving method that was more feasible for routine practice. Third, only one specific OCT angiography device was used in this study. Further research is needed to test the repeatability of the MLC findings in different devices. Last but not least, further histological studies are needed to confirm the specific cell types and their role in the diseased retina.

## Conclusions

In the current study, we observed MLC changes in living human retina in acute ischemic retinal stroke. Increased density and morphological changes in MLCs may indicate the aggregation and activation of MLCs after ischemia-reperfusion injury following acute artery occlusion. Moreover, MLCs characterized by en face OCT may serve as a tool to investigate neuroinflammation following artery ischemic stroke. The retina might be a potential window to investigate the pathophysiological changes related to the neurovascular unit in translational stroke research.

## Data availability statement

The raw data supporting the conclusions of this article will be made available by the authors, without undue reservation.

## Ethics statement

The studies involving human participants were reviewed and approved by Institutional Review Board of the Zhongshan Ophthalmic Center. The patients/participants provided their written informed consent to participate in this study.

## Author contributions

YZ and XZ: conception and design of the study. YZ, FW, LM, and YJ: data collection, analysis, and/or interpretation of the data. YZ and XZ: drafting the article. All authors: revising it critically for important intellectual content. XZ: final approval of the version to be published. All authors contributed to the article and approved the submitted version.

## Funding

This work was supported by the National Natural Science Foundation of China [Grant Number: 82070970].

## Conflict of interest

The authors declare that the research was conducted in the absence of any commercial or financial relationships that could be construed as a potential conflict of interest.

The reviewer DC declared a shared affiliation with the authors to the handling editor at the time of review.

## Publisher’s note

All claims expressed in this article are solely those of the authors and do not necessarily represent those of their affiliated organizations, or those of the publisher, the editors and the reviewers. Any product that may be evaluated in this article, or claim that may be made by its manufacturer, is not guaranteed or endorsed by the publisher.
